# Physical activity as a mediator of the impact of chronic conditions on quality of life in older adults

**DOI:** 10.1186/1477-7525-5-68

**Published:** 2007-12-19

**Authors:** Richard Sawatzky, Teresa Liu-Ambrose, William C Miller, Carlo A Marra

**Affiliations:** 1 Nursing Department, Trinity Western University, 7600 Langley, British Columbia, V2Y 1Y1, Canada; 2 Department of Physical Therapy, University of British Columbia, T325 2211 Wesbrook Mall, Vancouver, British Columbia, V6T 2B5, Canada; 3 Department of Occupational Science and Occupational Therapy, University of British Columbia, T325 2211 Wesbrook Mall, Vancouver, British Columbia, V6T 2B5, Canada; 4 GF Strong Rehabilitation Research Laboratory, University of British Columbia, T325 2211 Wesbrook Mall, Vancouver, British Columbia, V6T 2B5, Canada; 5 Faculty of Pharmaceutical Sciences, University of British Columbia, 2146 East Mall, Vancouver, British Columbia, V6T 1Z3, Canada; 6 Centre for Health Evaluation and Outcomes Sciences, Providence Health Care, St Paul's Hospital, 620B 1081 Burrard Street, Vancouver, B.C., V6Z 1Y6, Canada

## Abstract

**Background:**

Chronic conditions could negatively affect the quality of life of older adults. This may be partially due to a relative lack of physical activity. We examined whether physical activity mediates the relationship between different chronic conditions and several health outcomes that are important to the quality of life of older adults.

**Methods:**

The data were taken from the Canadian Community Health Survey (cycle 1.1), a cross-section survey completed in 2001. Only respondents who were 65 years or older were included in our study (*N *= 22,432). The Health Utilities Index Mark 3 (HUI3) was used to measure overall quality of life, and to measure selected health outcomes (dexterity, mobility, pain, cognition, and emotional wellbeing) that are considered to be of importance to the quality of life of older adults. Leisure-time physical activity was assessed by determining weekly energy expenditure (Kcal per week) based on the metabolic equivalents of self-reported leisure activities. Linear and logistic regression models were used to determine the mediating effect of leisure-time physical activity while controlling for demographic variables (age and sex), substance use (tobacco use and alcohol consumption), and obesity.

**Results:**

Having a chronic condition was associated with a relative decrease in health utility scores and a relative increase in mobility limitations, dexterity problems, pain, emotional problems (i.e., decreased happiness), and cognitive limitations. These negative consequences could be partially attributed to a relative lack of physical activity in older adults with a chronic condition (14% mediation for the HUI3 score). The corresponding degree of mediation was 18% for mobility limitations, 5% for pain, and 13% for emotional wellbeing (statistically significant mediation was not observed for the other health attributes). These values varied with respect to the different chronic conditions examined in our study.

**Conclusion:**

Older adults with chronic conditions are less likely to engage in leisure-time physical activities of at least 1,000 Kcal per week, and this association partially accounts for some negative consequences of chronic conditions, including mobility limitations, pain, and emotional problems. These findings provide support for health promotion programs that facilitate or encourage increased leisure-time physical activity in older people with chronic conditions.

## Background

A chronic condition can be defined as a medical condition that is slow in its progress and long in its continuance. More than 80% of Canadians aged 65 and older report having at least one chronic condition [1]. Chronic conditions contribute to disability via physical impairments and functional limitations and consequently diminish quality of life in older adults. In older adults, chronic conditions have been associated with an increased risk for a variety of secondary health issues including medical conditions, such as disuse osteoporosis concomitant to sustaining a stroke, and psychosocial challenges, such as those related to depression and pain [2-4]. Chronic conditions also increase the costs of health care and long-term care [5]. Thus, the increased prevalence of chronic conditions in the aging population poses a significant challenge to society and the health care system.

Physical activity is a proven but remarkably underused health promotion modality [6]. Evidence has shown that regular physical activity contributes to healthy aging by preventing disability, morbidity, and mortality in older adults [7]. It has been demonstrated that physical activity decreases the likelihood of dying with disability almost two-fold when comparing those most physically active to those who were sedentary [8]. A graded, inverse relationship between total physical activity and mortality has been identified [9]. Regular physical activity can modify the severity or the progression of chronic conditions, thereby reducing both morbidity and mortality associated with chronic conditions [7]. Physical activity has various psychological and social benefits. For example, studies have shown that exercise alleviates depression [10], and provides additional therapeutic benefits beyond those resulting from psychotherapy [11] and the use of psychotropic medications [12,13]. Despite its many benefits, physical activity participation declines progressively with age [14], particularly among older adults who have chronic conditions.

Studies have demonstrated that physical activity can improve quality of life in adults with chronic conditions [15,16]. These associations have typically been examined with respect to a particular chronic condition, such as arthritis. However, it is unclear to what degree the negative impact of chronic conditions on quality of life and important health outcomes in older adults can be attributed to a lack of physical activity. It is also unclear whether this hypothesized mediating effect of physical activity is consistent with respect to different chronic conditions. This information is vital to understanding the role of physical activity in promoting quality of life in older adults.

The analytical objectives for this study are to: 1) examine the degree to which the negative impact of chronic conditions on quality of life and various important health outcomes (e.g., emotional problems, mobility limitations, pain, emotional wellbeing, and cognitive limitations) in older adults could be attributed to a lack of physical activity; and 2) examine whether the hypothesized mediating effect of physical activity is consistent with respect to some of the most prevalent chronic conditions in older adults (including musculoskeletal disorders, cardiovascular disorders, respiratory disorders, diabetes, urinary or bowel disorders, and strokes). We specifically hypothesized that those older adults who have a chronic condition but who maintained the recommended amount of physically activity of 1,000 Kcal per week would experience better health outcomes than those who are physically inactive.

## Methods

The data were obtained from the Canadian Community Health Survey (CCHS) cycle 1.1 (Statistics Canada): a multi-cycle cross-sectional health survey of the Canadian population that contains information about chronic conditions, various health outcomes, health resource utilization, socio-demographics, and physical activity [17]. The sampling strategy included a stratified cluster design (83% of total sample) to obtain proportional geographic and socio-economic representation of dwelling units across the 136 health regions in Canada. This sampling strategy was supplemented with a random digit dialing approach (10% of total sample) and a list frame of telephone numbers (7% of the total sample). This resulted in a total sample of 130,880 respondents who were all contacted by telephone to complete the survey. The national non-response rate was estimated at 20.0% [17]. People living in Indian Reserves, the Canadian Forces Bases, some remote areas, and people who did not dwell in a household as defined by Statistics Canada were not included. For this study, we utilized the data from respondents aged 65 years and older (*N = *24,281).

The data were collected by Statistics Canada under the authority of the Statistics Act. Access to the data was granted by Statistics Canada based on a peer-reviewed proposal for this study. The researchers did not have access to any identifying information so that anonymity of the respondents was protected. The opinions expressed here do not represent the views of Statistics Canada.

### Classification of chronic conditions

**Figure 1:**
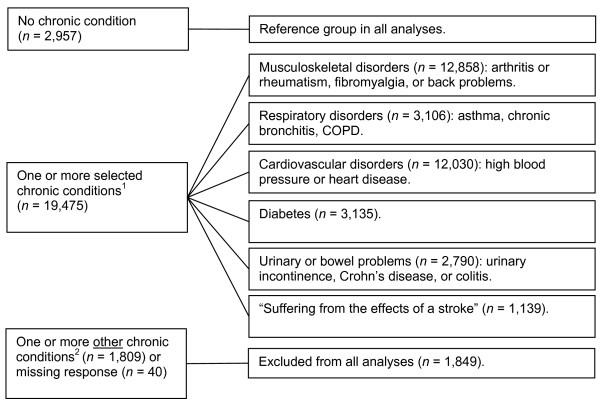
**Classification of chronic conditions in the sample of older adults**. Notes:*N *= 24,281. ^1 ^The following selected chronic conditions were included: asthma, fibromyalgia, arthritis or rheumatism, back problems, high blood pressure, chronic bronchitis, emphysema or chronic obstructive pulmonary disease (COPD), diabetes, heart disease, cancer, stroke, urinary incontinence, Crohn's disease or colitis, Alzheimer's disease or other dementia, Parkinson's disease, multiple sclerosis. ^2 ^Excluded from all analyses were older adults who did not have any of the above chronic conditions but who did report having food or other allergies, migraine headaches, epilepsy, stomach or intestinal ulcers, cataracts, glaucoma, a thyroid condition, chronic fatigue syndrome, chemical sensitivities, or any other long-term chronic condition diagnosed by a health care professional.

The respondents were asked to indicate whether they had a disease or another health condition diagnosed by a health professional that had lasted, or was expected to last, 6 months or more. These data were used to classify the older adults into the following overlapping groups based on those chronic conditions that are similar with respect to the predominant body systems involved: 1) respiratory disorders (asthma, chronic bronchitis, emphysema or chronic obstructive pulmonary disease), 2) musculoskeletal disorders (arthritis, fibromyalgia or back problems), 3) cardiovascular disorders (high blood pressure or heart disease), 4) diabetes, 5) urinary or bowel problems (urinary incontinence, Crohn's disease or colitis), and 6) those who were "suffering the effects of a stroke". Older adults with cancer, Alzheimer's disease or another form of dementia, Parkinson's disease, or multiple sclerosis were also included in our analyses. However, older adults who did not have any of the above chronic conditions but who did report having another chronic condition were not included (*n *= 1,809). Some chronic conditions, such as food or other allergies, cataracts, glaucoma, and thyroid conditions were not considered because their impact on quality of life, as measured by the Health Utilities Index [18], has previously shown to be indiscernible or mild in older adults [19]. Migraine headaches and epilepsy were not considered because their sporadic nature did not lend itself well to a cross-sectional analysis. We first compared the older adults who had one or more of the selected chronic conditions (*n *= 19,475) to those who reported having no chronic condition (*n *= 2,957), and we subsequently repeated these analyses for each of the above chronic condition groups (see Figure 1; the corresponding sample sizes for the chronic condition groups after listwise deletion are shown in Table 1).

**Table 1:** Description of the chronic condition groups

			Chronic condition groups					
			
Category	No chronic condition (*n *= 2,639)	One or more Chronic conditions (*n *= 17,314)	Respiratory disorders (*n *= 2,722)	Musculo- skeletal disorders (*n *= 11,473)	Cardio- vascular disorders (*n *= 10,741)	Diabetes (*n *= 2,754)	Urinary or bowel disorders (*n *= 2,399)	Stroke (*n *= 894)
Activity								
≥ 1,000 Kcal/week	35.1%	25.8%	20.7%	24.8%	24.7%	24.3%	20.0%	17.2%
Age								
65 – 74 yrs	69.7%	58.3%	58.1%	57.4%	56.3%	61.2%	48.9%	43.1%
75 – 79 yrs	17.1%	21.2%	22.2%	21.3%	22.5%	21.8%	21.9%	26.4%
> 84 yrs	13.2%	20.5%	19.7%	21.3%	21.2%	17.0%	29.2%	30.5%
Sex								
Female	46.2%	59.6%	56.9%	65.0%	59.1%	50.8%	68.3%	50.7%
Smoking								
Yes	16.7%	11.6%	15.6%	11.8%	10.1%	9.0%	11.6%	11.5%
Alcohol use								
Does not use alcohol	28.0%	34.4%	37.8%	34.5%	36.0%	47.1%	37.2%	44.8%
< 2 times/month	18.2%	21.7%	20.3%	22.3%	21.7%	21.7%	24.6%	19.8%
2 to 3 times/month	14.4%	12.9%	11.9%	12.8%	12.8%	10.3%	12.5%	10.8%
> 3 times/month	39.4%	31.0%	30.1%	30.4%	29.5%	21.0%	25.7%	24.7%
Obesity								
BMI < 18.5	55.6%	42.5%	42.3%	40.6%	39.7%	30.9%	41.8%	46.2%
BMI 18.5 – 25	2.8%	3.1%	4.4%	3.1%	2.6%	1.2%	3.3%	4.8%
BMI ≥ 25	41.7%	54.4%	53.4%	56.2%	57.8%	67.9%	55.0%	49.0%

Notes: *N *= 19,953, including those older adults who had no chronic conditions or who had one of the selected chronic conditions and for whom there was no missing data for any of the variables in our analyses.

### Dependent variables

The dependent variables of interest were various health outcomes that are generally considered to be of importance to quality of life. The Health Utility Index Mark 3 (HUI3) [18,20,21] was used in the CCHS for the measurement of these health outcomes. This instrument consists of 31 questions pertaining to eight health attributes that represent limitations associated with hearing, vision, speech, cognition, mobility, dexterity, pain, and emotional wellbeing (happiness). Utility weights for several health states were derived from the preferences obtained from a community sample of 504 adults in the city of Hamilton, Ontario, Canada [22]. Multi-attribute theory was used to calculate a total health utility score that can range from – 0.36 ("most disabled") to 1.00 ("perfect health") [22].

The HUI3 was also used to examine the impact of chronic conditions and physical activity on several distinct health attributes (including cognition, mobility, dexterity, pain and emotional wellbeing). The guidelines provided by the instrument developers were followed to concatenate the HUI3 questions to obtain ordinal summary scores for these attributes. The resulting ordinal variables were collapsed into dichotomous variables as shown in Table 2.

**Table 2:** Bivariate associations among the HUI3 attributes having a chronic condition

Variable	No chronic condition (*n *= 2,639)	One or more chronic conditions (*n *= 17,314)	Odds ratio^1^ (95% CI)
Mobility			
No difficulty walking (referent)	97.1%	84.0%	1.00
Difficulty walking or unable to walk	2.9%	16.0%	6.4 (4.7 – 8.7)
Dexterity			
Full use of hands and fingers (referent)	99.8%	97.8%	1.00
Any limitation in the use of hands or fingers	0.2%	2.2%	9.6 (3.7 – 24.9)
Emotion			
Happy or somewhat happy (referent)	98.7%	94.7%	1.00
Somewhat or very unhappy	1.3%	5.4%	4.3 (2.7 – 6.8)
Cognition			
No cognitive limitations (referent)	80.6%	66.7%	1.00
Any cognitive limitations	19.4%	33.3%	2.1 (1.8 – 2.4)
Pain			
Free of pain or discomfort (referent)	94.9%	69.3%	1.00
Mild, moderate, or severe pain	5.1%	30.7%	8.3 (6.2 – 11.0)

Notes: *N *= 19,953.

^1 ^Bivariate logistic regression was used to calculate the confidence intervals.

### Independent variables

The respondents were asked about the frequency and amount of time that they engaged in physical leisure activities over the past three months (e.g., specific sports, gardening, exercise classes, etc.). A score for leisure-time physical activity was obtained by calculating weekly energy expenditure (kilocalories (Kcal) per week) based on the metabolic equivalents for each of the self-reported leisure activities [23]. We used the guidelines provided in the US Surgeon General's 1996 report as the basis for collapsing this variable so as to specifically compare those who had an energy expenditure of less than 1,000 Kcal per week to those who met the minimally recommended 1,000 Kcal of weekly energy expenditure [24].

Tobacco use, alcohol consumption, and obesity were included as additional health-related covariates in our analyses. Older adults who reported smoking daily or occasionally at the time of the survey were compared to those who did not smoke. Alcohol consumption was assessed based on responses to the question "During the past 12 months, how often did you drink alcoholic beverages?" This variable was collapsed into four categories: 1) no alcohol consumption, 2) between one and three times a month, 3) once a week, and 4) more than once a week. The body mass index (BMI) was used to classify the older adults as being of normal weight (BMI ≥ 18.5 and < 25), underweight (BMI < 18.5), or overweight or obese (≥ 25). The respondent's age and sex were included as demographic covariates.

### Analytical approach

**Figure 2:**
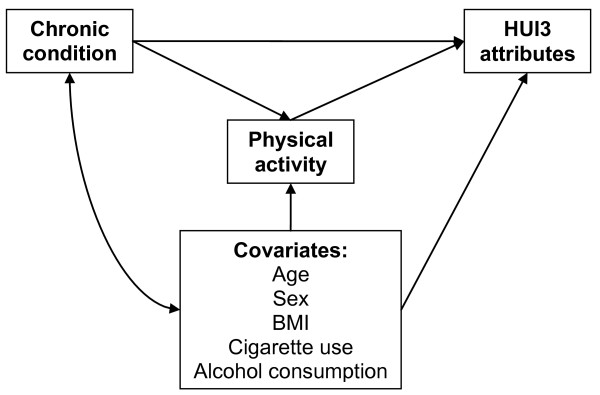
Heuristic diagram of hypothesized relationships.

We used ordinary least squares regression to estimate the relationships between having a chronic condition, physical activity, and the HUI3 score while controlling for the covariates mentioned above. As shown in Figure 2, the HUI3 score was regressed on the chronic condition variable, and physical activity was specified as a mediator of this relationship. The Pratt-Index (*d*) [25] was used to partition the *R-*square so as to determine the relative importance of the variables explaining the HUI3 score. This index was calculated by multiplying the standardized regression coefficients by the corresponding correlations and dividing that value by the *R*-square. Thus, the Pratt-Index value signifies the proportion of the *R*-square that is attributable to each of the variables in the model. We subsequently used binary logistic regression to examine the mediating effects of leisure-time physical activity independently for specific HUI3 attributes. The fit of the logistic models was assessed based on the likelihood ratio chi-square and the likelihood ratio *R*^2 ^(also known as McFadden's *R*^2^) [26].

The degree of mediation was determined by calculating the indirect effect as the product of the coefficients of the relationships between the HUI3 attributes and physical activity and having a chronic condition [27]. The standard error for the indirect effect was estimated using the delta method, which is similar to the approach of variance estimation used in the Sobel's test for mediating effects [28]. A simulation study by MacKinnon and Dwyer showed that the delta method led to accurate estimates of indirect effects and their standard errors when using binary data [28]. We followed their recommendations to evaluate the degree of mediation as the percentage of the total effect that could be attributed to the indirect effect.

The SAS 9.1 software package [29] was used to obtain the maximum likelihood estimates for each of the models. The bootstrapped sampling weights provided by Statistics Canada were used to obtain parameter estimates and their standard errors based on 500 replications of each model. All models were estimated using listwise deletion resulting in the exclusion of 2,479 (11.1%) respondents due to missing responses for one or more of the analysis variables. The parameter estimates were compared to those based on full information maximum likelihood estimation (FIML) (available in the Mplus 4.2 [30] software package) by using all available data to assess whether the estimates may have been biased by non-random missing data patterns (*n *= 21,736; excluding 696 (3.1%) respondents who did not provide any information regarding their HUI3 scores or any of the explanatory variable) [31,32].

## Results

### Sample description and bivariate associations

Most of the older adults (79%) had at least one of the chronic conditions that were considered in our study, 8% had a chronic condition other than the ones that were considered in our study, and 13% had no chronic condition (Figure 1). Only 25% of the older adults achieved the minimally recommended activity level of 1,000 Kcal per week (64% did not achieve the recommended activity level and 11% did not answer some or all questions about their leisure-time physical activity). Descriptive findings pertaining to each of the chronic condition groups are shown in Table 1.

The distribution of the HUI3 score was negatively skewed with a mean of 0.79 (SD = 0.25) and a median of 0.91 (*N *= 19,953). With respect to specific HUI3 attributes, most older adults reported having no limitations in cognition (69%), mobility (86%), and dexterity (98%). In addition, 73% reported having no pain, and 95% reported being happy or somewhat happy in life.

**Figure 3:**
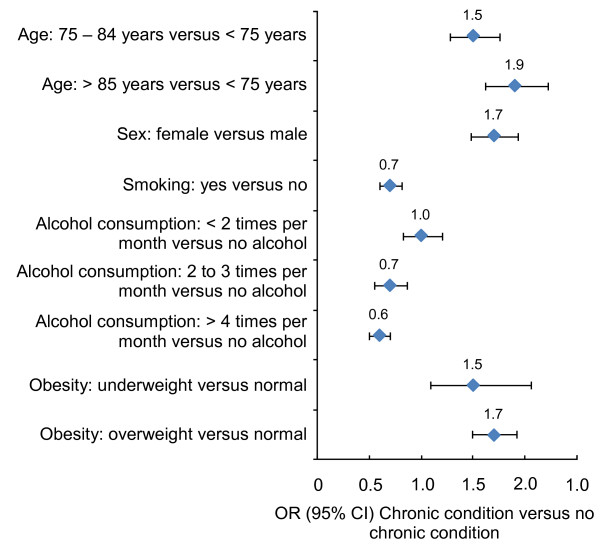
**Odds ratios for covariates**. Notes: *N = *19,953.

**Figure 4:**
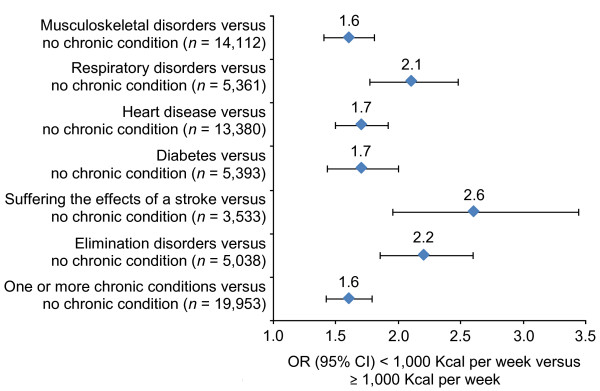
Odds ratios for physical activity in the chronic condition subsamples.

Those who had a chronic condition had relatively lower scores for each of the HUI3 attributes in comparison to those who had no chronic condition (Table 2). At the time of the survey, they were also less likely to have used tobacco, less likely to have consumed alcohol and more likely to be overweight (Figure 3). Fewer older adults who had a chronic condition achieved the recommended physical activity level of 1,000 Kcal per week relative to those older adults who did not have a chronic condition. The corresponding odds ratio (OR) in the overall sample was 1.6 (95% CI = 1.5 – 1.8), and the ORs ranged from 1.6 to 2.6 in the chronic condition subsamples (Figure 4).

### Multivariate analysis results

The F-test of model fit for the variables explaining the total HUI3 score was statistically significant (F (11, 19,941) = 254, *p *< 0.01, *R*^2 ^= 12%) (Table 3). The HUI3 score was predominantly explained by differences in age (Pratt Index = 0.35), having a chronic condition (Pratt Index = 0.28), leisure-time physical activity (Pratt Index = 0.19), and alcohol consumption (Pratt Index = 0.15). Although the effects of the other variables were statistically significant, they only accounted for a total of 2% of the explained variance. Relatively lower HUI3 scores were observed for those who had a chronic condition (b = -0.13, *p *< 0.01), and relatively higher HUI3 scores were observed for those who were physically active (b = 0.07, *p *< 0.01) after controlling for differences in age, gender, tobacco use, alcohol consumption, and obesity.

**Table 3:** Regression model results in the full sample

	Dependent variables				
	
Variables	HUI total score b(se)	Mobility OR (95% CI)	Pain OR (95% CI)	Emotion OR (95% CI)	Physical activity OR (95% CI)
Physical activity (referent = ≥ 1,000 Kcal/week)					
< 1,000 Kcal/week	-0.07 (0.00)	3.6 (4.3 – 3.0)	1.5 (1.7 – 1.3)	2.2 (1.6 – 3.0)	-
Age (referent = 65 – 74 yrs)					
75 – 84 yrs	-0.04 (0.01)	2.0 (1.8 – 2.4)	1.0 (0.9 – 1.2)	1.1 (0.8 – 1.5)	1.6 (1.4 – 1.9)
> 84 yrs	-0.12 (0.01)	4.9 (4.2 – 5.6)	1.1 (1.0 – 1.2)	1.3 (1.0 – 1.6)	2.3 (2.0 – 2.6)
Sex (referent = male)					
Female	0.02 (0.01)	0.9 (0.8 – 1.0)	1.3 (1.2 – 1.4)	0.9 (0.7 – 1.1)	2.3 (2.1 – 2.6)
Smoking status (referent = does not smoke)					
Smokes daily or occasionally	-0.04 (0.01)	1.5 (1.2 – 1.8)	1.2 (1.1 – 1.4)	1.8 (1.4 – 2.3)	2.0 (1.7 – 2.3)
Alcohol use (referent = does not use alcohol)					
Less than two times/month	0.03 (0.01)	0.9 (0.8 – 1.0)	0.9 (0.8 – 1.0)	0.7 (0.5 – 1.0)	0.8 (0.7 – 1.0)
Two or three times/month	0.06 (0.01)	0.6 (0.5 – 0.7)	0.8 (0.6 – 0.9)	0.5 (0.3 – 0.8)	0.7 (0.6 – 0.8)
Four or more times/month	0.07 (0.01)	0.6 (0.5 – 0.7)	0.7 (0.6 – 0.8)	0.4 (0.3 – 0.6)	0.6 (0.5 – 0.6)
Obesity (referent = between 18.5 and 25)					
Less than 18.5	-0.06 (0.02)	1.6 (1.2 – 2.2)	1.4 (1.1 – 1.8)	2.1 (1.4 – 3.2)	3.7 (2.6 – 5.4)
More than or equal to 25	-0.01 (0.00)	1.5 (1.3 – 1.7)	1.2 (1.1 – 1.3)	0.9 (0.8 – 1.2)	1.0 (0.9 – 1.1)
Chronic condition(s) (referent = no chronic conditions)					
One or more chronic conditions	-0.13 (0.00)	5.1 (3.8 – 7.0)	7.6 (5.7 – 10.1)	4.0 (2.5 – 6.3)	1.3 (1.2 – 1.5)
Indirect effect^1^	-0.02 (0.01)	1.4 (1.2 – 1.7)	1.1 (1.1 – 1.2)	1.2 (1.1 – 1.2)	-
% mediated by physical activity^2^	14%	18%	5%	13%	-

*R*^2 ^(LR *R*^2^)	12%	(15%)	(6%)	(6%)	(8%)
Likelihood ratio chi-square (Df = 11)	n/a	2,358.59	1,444.94	423.90	1,878.80

Notes: *N *= 19,953, including those older adults who had no chronic conditions or one of the selected chronic conditions and for whom there was no missing data for any of the variables in our analyses. Only the results for the HUI3 attributes with statistically significant indirect effects (*p *< 0.01) are shown. The reference groups for mobility, pain, and emotion are the same as in Table 2.

^1 ^The indirect effect of having a chronic condition versus no chronic condition as mediated by physical activity.

^2 ^Percentage of the total effect of having a chronic condition that is attributed to the mediating role of physical activity after controlling for the covariates (based on the unexponentiated regression weights).

The relationship between having a chronic condition and leisure-time physical activity was examined to determine whether physical activity mediated the negative impact of having a chronic condition on the HUI3 score. The likelihood ratio test of global model fit for variables explaining the physical activity was statistically significant (LR χ^2^_(10) _= 1,878.80, *p *< 0.01, LR *R*^2 ^= 8%). Physical activity was significantly associated with differences in age, alcohol consumption, smoking status, and having a chronic condition (last column Table 3). Thus, the negative impact of having a chronic condition was partially mediated by physical activity (14% mediation), and the corresponding indirect effect was statistically significant (*p *< 0.01) after controlling for the covariates (Table 3). The indirect effects for the HUI3 attributes were statistically significant for mobility limitations, pain, and emotional wellbeing (Table 3). The average percentages of the total impact of having a chronic condition that could be attributed to the mediating role of physical activity were 18% for mobility challenges, 13% for emotional problems, and 5% for pain. We did not observe statistically significant (*p <*0.01) indirect effects for dexterity problems and cognition.

The above associations were examined independently in each of the six chronic condition subsamples (Table 4). Having a chronic condition was significantly associated with a relative increase in mobility limitations, pain, and emotional problems in all chronic condition subsamples. The adjusted ORs for the effect of having a chronic condition on leisure-time physical activity when controlling for the covariates ranged from 1.3 (95% CI = 1.1 – 1.5) for older adults with a musculoskeletal disorder to 2.1 (95% CI = 1.6 – 2.8) for older adults who suffered the consequences of a stroke. Those who were more physically active reported relatively fewer mobility limitations (OR ranging from 2.6 to 3.9) and less pain (OR ranging from 1.3 to 2.0) in the chronic condition subsamples (Table 4). Increased physical activity was also associated with a relative increase in emotional wellbeing and relatively fewer cognitive problems and dexterity limitations in some of the chronic condition subsamples. The indirect effects were statistically significant for mobility limitations (ranging from 16% in the musculoskeletal disorders subsample to 27% in the respiratory disorders subsample) in all of the chronic condition subsamples (last column Table 4). Similar results with respect to the magnitude of the parameters were obtained when these analyses were replicated using FIML.

**Table 4:** Odds ratios and % mediation for selected HUI3 attributes in the chronic condition subsamples

	HUI3 attributes (dependent variables)				
	
Independent variables	Dexterity OR (95% CI)	Emotional wellbeing OR (95% CI)	Cognition OR (95% CI)	Pain OR (95% CI)	Mobility OR (95% CI)
Musculoskeletal disorders versus no chronic condition (*n *= 14,112)^1^	11.0 (4.3 – 28.5)	4.7 (2.9 – 7.6)	2.2 (2.0 – 2.5)	12.0 (9.0 – 16.1)	6.6 (4.8 – 9.0)
Physical activity < 1,000 Kcal/week^2^	1.5 (1.0 – 2.3)	2.3 (1.6 – 3.3)	1.1 (1.0 – 1.3)	1.4 (1.2 – 1.7)	3.7 (3.0 – 4.5)
% mediation^3^	5%	13%*	4%	4%*	16%*

Respiratory disorders versus no chronic condition (*n *= 5,361)^1^	10.4 (3.7 – 28.9)	5.0 (3.0 – 8.1)	2.2 (1.8 – 2.6)	10.7 (8.0 – 14.5)	7.6 (5.4 – 10.7)
Physical activity < 1,000 Kcal/week^2^	0.8 (0.4 – 1.5)	2.0 (0.9 – 4.5)	1.2 (1.0 – 1.5)	1.4 (1.0 – 1.8)	3.9 (2.5 – 6.0)
% mediation^3^	0%	20%	13%	7%	27%*

Cardiovascular disorders versus no chronic condition (*n *= 13,380)^1^	7.8 (3.0 – 20.0)	4.0 (2.5 – 6.4)	1.9 (1.7 – 2.2)	7.2 (5.4 – 9.5)	5.6 (4.1 – 7.7)
Physical activity < 1,000 Kcal/week^2^	1.4 (0.9 – 2.2)	2.1 (1.4 – 3.2)	1.2 (1.0 – 1.3)	1.6 (1.3 – 1.9)	3.3 (2.6 – 4.1)
% mediation^3^	5%	16%*	7%	8%*	19%*

Diabetes versus no chronic condition (*n *= 5,393)^1^	10.6 (4.3 – 26.5)	5.0 (3.0 – 8.5)	1.9 (1.6 – 2.3)	7.1 (5.2 – 9.7)	6.6 (4.8 – 9.2)
Physical activity < 1,000 Kcal/week^2^	1.2 (0.5 – 3.1)	1.9 (0.8 – 4.1)	1.2 (1.0 – 1.5)	1.7 (1.3 – 2.3)	3.5 (2.3 – 5.3)
% mediation^3^	3%	13%	10%	10%*	21%*

"Suffering the effects of a stroke" versus no chronic condition (*n *= 3,533)^1^	24.9 (7.9 – 78.1)	9.4 (5.1 – 17.5)	3.4 (2.7 – 4.3)	12.4 (8.7 – 17.7)	18.2 (12.7 – 26.1)
Physical activity < 1,000 Kcal/week^2^	0.6 (0.2 – 2.4)	1.2 (0.4 – 3.6)	1.0 (0.8 – 1.4)	1.3 (0.8 – 2.0)	2.6 (1.5 – 4.6)
% mediation^3^	0%	5%	3%	7%	20%*

Urinary or bowel disorders versus no chronic condition (*n *= 5,038)^1^	15.3 (5.8 – 40.5)	7.7 (4.5 – 13.1)	3.1 (2.6 – 3.8)	14.4 (10.5 – 19.7)	9.9 (7.1 – 13.9)
Physical activity < 1,000 Kcal/week^2^	1.0 (0.6 – 1.8)	1.2 (0.7 – 2.1)	1.1 (0.9 – 1.4)	2.0 (1.5 – 2.7)	2.9 (2.0 – 4.2)
% mediation^3^	0%	4%	6%	12%*	20%*

All odds ratios are adjusted for age, sex, cigarette use, alcohol consumption, and obesity. The reference groups for the HUI3 attributes are the same as in Table 2.

^1 ^Referent = no chronic condition.

^2 ^Referent = ≥ 1,000 Kcal/week.

^3 ^Percentage of the total effect that is attributable to the mediating effect of physical activity.

* Statistically significant indirect effects (*p *< 0.01).

## Discussion

To our knowledge, this is the first study that has specifically examined degree to which the negative impact of chronic conditions on quality of life in older adults could be attributed to a lack of physical activity. The results suggest that physical activity partially mediates the impact of chronic conditions on several health outcomes that are important to quality of life. Physical activity of at least 1,000 Kcal per week was associated with relatively fewer mobility limitations, reduced pain, and greater emotional wellbeing (i.e., happiness). The clinical relevance of the mediating role of physical activity can be inferred by comparing the magnitude of the indirect effect to that of the total effect, which indicated up to 27% mediation for mobility limitation, up to 12% mediation for pain, and up to 16% mediation for emotional wellbeing. These findings concur with those of other studies. For example, adequate physical activity was associated with a significant reduction in the number of days of poor physical and mental health status in adults with arthritis [15].

The US Center for Disease Control and the American College of Sports Medicine guidelines [33] recommended that individuals should engage in 30 minutes or more of moderate-intensity physical activity on a daily basis (equivalent to approximately 1,400 Kcal/week) while the US Surgeon General's 1996 report classified moderate physical activity as more than 1,000 Kcal/week [24]. We found a low level of participation in leisure-time physical activity regardless of chronic disease status among older Canadians. Specifically, only 35% of older adults without any chronic condition and 26% of those with one or more chronic conditions met the 1,000 Kcal/week criterion.

Epidemiological data have established that physical inactivity decreases the incidence of at least 17 unhealthy conditions, most of which are chronic conditions or risk factors [7]. Our study further elucidates the importance of physical activity for older adults who have a chronic condition. We found that older adults with chronic conditions who were physical active (i.e., leisure-time physical activity of at least 1,000 Kcal per week) reported better health outcomes related to mobility, pain, and emotional wellbeing than those who were physical inactive. Leisure-time physical activity likely mediates the negative association between chronic conditions and these specific self-reported health outcomes in older adults by: 1) maintaining or augmenting physiological functions (e.g., prevention of sarcopenia); 2) reducing the likelihood of acquiring additional chronic conditions; 3) delaying the progression of current chronic condition(s); and 4) improving mental health and sense of wellbeing. In sum, physical activity beneficially affects the human body in a multifactorial manner.

Regular physical activity not only directly promotes mobility in older adults via mechanisms such as improved muscle strength and postural balance but also indirectly by, for example, reducing the risk for falls and fractures [34,35]. Maintaining the capacity for independent mobility and living is important to older adults and contributes to their general sense of emotional wellbeing [36,37]. Physical activity can enhance emotional wellbeing via increases in: 1) beta endorphins; 2) the availability of brain neurotransmitters (e.g. serotonin); and 3) self-efficacy [38]. In addition, physical activity may mediate the negative association between chronic conditions and health outcomes by reducing the likelihood of acquiring additional chronic conditions and delaying the progression of current chronic condition(s). Most prevalent chronic conditions have an association with physical inactivity, and a number of risk factors for chronic conditions are precipitated by physical inactivity (e.g., obesity [39] and insulin resistance [40]).

Unfortunately, individuals with chronic conditions are at the highest risk of physical inactivity [24] – placing these individuals at greater risk for acquiring additional chronic conditions. According to Booth and coworkers [7], physical inactivity is the key environmental factor contributing to the substantial increase in the incidence of chronic conditions in the latter part of the 20^th ^century. Thus, physical activity can prevent the onset of chronic conditions. Our findings suggest that physical activity could also be beneficial for older adults who already have one or more chronic conditions. These findings provide further support for health promotion programs that facilitate or encourage increased leisure-time physical activity in older people with chronic conditions.

In this study, physical activity is measured as the time spent performing leisure-time activities. Despite the comprehensive nature of this information, daily activities performed by individuals are not represented in these data and therefore physical activity was conservatively estimated. In addition, some respondents may not have been able to accurately recall all their leisure-time physical activities for a period of three months. This may explain why the magnitude of the mediation effect that we observed in this study was smaller than we had anticipated. We specifically expected that the OR for the association between having a chronic condition and physical activity would have been larger. Non-response bias may also have contributed to these results (e.g., older adults with severe physical or mental health problems may have been less likely to complete the survey).

A few other limitations should be noted. Although the relationships were specified to examine the mediating effects of physical activity, the direction of these relationships could also operate in the reverse. The cross-sectional nature of the data does not allow us to confirm claims pertaining to the causality of these relationships. It seems just as likely that poor ambulation will lead to a decrease in physical activity which could lead to a variety of chronic conditions. In addition, the utility weights for the HUI3 may not be generalizable considering that they are based on a community sample of 504 adults in the city of Hamilton, Ontario, Canada [22]. Nevertheless, these weights were only used for calculating the total HUI3 scores; they were not used to measure each of the health attributes which were included as binary variables in our analyses. And, there is a lack of independence in our categories of chronic conditions. For instance individuals who have had a stroke are likely to have cardiovascular conditions as well. Finally, some chronic conditions that may impact quality of life in older adults (e.g., epilepsy and migraine headaches) were not included in our analyses.

## Conclusion

We observed that older adults with chronic conditions are less likely to engage in leisure-time physical activities of at least 1,000 Kcal per week, and that association partially accounts for some negative consequences of chronic conditions, including mobility limitations, pain, and emotional problems. We recommend that increased attention be paid to physical activity as a potential health promotion modality for older adults with chronic conditions. Further studies are needed to determine the particular types of physical activities that are most beneficial for older adults with specific chronic conditions.

## Abbreviations

BMI Body mass index

CI Confidence interval

CCHS Canadian Community Health Survey

FIML Full information maximum likelihood

HUI3 Health Utilities Index (Mark 3)

Kcal Kilocalories

LR Likelihood ratio

OR Odds ratio

SD Standard deviation

## Competing interests

The author(s) declare that they have no competing interests.

## Authors' contributions

RS designed and carried out the statistical analyses and drafted the manuscript. TLA assisted with the interpretation of the results and contributed to the writing and editing of multiple drafts. WCM conceived and designed the project, obtained funding, assisted with the interpretation of the results and contributed to the writing and editing of multiple drafts. CAM was involved in the design, assisted in the interpretation of results and edited multiple drafts of the manuscript. All authors read and approved the final manuscript.
